# Supplementary material for: Recent Advance Analysis of Recovery in Hospitalized People with COVID-19: A Systematic Review
Source: Int J Environ Res Public Health. 2022 Nov 7;19(21):14609. doi: 10.3390/ijerph192114609 (PMC9655961; doi:10.3390/ijerph192114609)
Supplement: Supplementary file 1 [file ijerph-19-14609-s001.zip › ijerph-2009676-supplementary.pdf]

SUPPLEMENTARY MATERIAL

Table S1. Search strategy

**Search strategy**	**Date**
OVID (MEDLINE, EMBASE, COCHRANE, AMED)<br>1. Longitudinal stud*.mp. [mp=ab, hw, ti, tx, kw, ct, ot, sh, tn, dm, mf, dv, fx, dq, nm, kf, ox, px, rx, ui, sy]<br>2. Prospective Stud*.mp. [mp=ab, hw, ti, tx, kw, ct, ot, sh, tn, dm, mf, dv, fx, dq, nm, kf, ox, px, rx, ui, sy]<br>3. Follow-up stud*.mp. [mp=ab, hw, ti, tx, kw, ct, ot, sh, tn, dm, mf, dv, fx, dq, nm, kf, ox, px, rx, ui, sy]<br>4. Prognosis.mp. [mp=ab, hw, ti, tx, kw, ct, ot, sh, tn, dm, mf, dv, fx, dq, nm, kf, ox, px, rx, ui, sy]<br>5. Survival.mp. [mp=ab, hw, ti, tx, kw, ct, ot, sh, tn, dm, mf, dv, fx, dq, nm, kf, ox, px, rx, ui, sy]<br>6. Survival analysis.mp. [mp=ab, hw, ti, tx, kw, ct, ot, sh, tn, dm, mf, dv, fx, dq, nm, kf, ox, px, rx, ui, sy]<br>7. Logistic model*.mp. [mp=ab, hw, ti, tx, kw, ct, ot, sh, tn, dm, mf, dv, fx, dq, nm, kf, ox, px, rx, ui, sy]<br>8. Proportional hazards model*.mp. [mp=ab, hw, ti, tx, kw, ct, ot, sh, tn, dm, mf, dv, fx, dq, nm, kf, ox, px, rx, ui, sy]<br>9. Disease Progression.mp. [mp=ab, hw, ti, tx, kw, ct, ot, sh, tn, dm, mf, dv, fx, dq, nm, kf, ox, px, rx, ui, sy]<br>10. Life Table*.mp. [mp=ab, hw, ti, tx, kw, ct, ot, sh, tn, dm, mf, dv, fx, dq, nm, kf, ox, px, rx, ui, sy]<br>11. cohort stud*.mp. [mp=ab, hw, ti, tx, kw, ct, ot, sh, tn, dm, mf, dv, fx, dq, nm, kf, ox, px, rx, ui, sy]<br>12. prognos*.mp. [mp=ab, hw, ti, tx, kw, ct, ot, sh, tn, dm, mf, dv, fx, dq, nm, kf, ox, px, rx, ui, sy]<br>13. course.mp. [mp=ab, hw, ti, tx, kw, ct, ot, sh, tn, dm, mf, dv, fx, dq, nm, kf, ox, px, rx, ui, sy]<br>14. inception.mp. [mp=ab, hw, ti, tx, kw, ct, ot, sh, tn, dm, mf, dv, fx, dq, nm, kf, ox, px, rx, ui, sy]<br>15. 1 or 2 or 3 or 4 or 5 or 6 or 7 or 8 or 9 or 10 or 11 or 12 or 13 or 14<br>16. COVID-19.mp. [mp=ab, hw, kw, ti, ot, tx, ct, sh, tn, dm, mf, dv, fx, dq, nm, kf, ox, px, rx, an, ui, sy]<br>17. Coronavirus disease.mp. [mp=ab, hw, kw, ti, ot, tx, ct, sh, tn, dm, mf, dv, fx, dq, nm, kf, ox, px, rx, an, ui, sy]<br>18. nCov.mp. [mp=ab, hw, kw, ti, ot, tx, ct, sh, tn, dm, mf, dv, fx, dq, nm, kf, ox, px, rx, an, ui, sy]<br>19. 19nCov.mp. [mp=ab, hw, kw, ti, ot, tx, ct, sh, tn, dm, mf, dv, fx, dq, nm, kf, ox, px, rx, an, ui, sy]<br>20. novel coronavirus.mp. [mp=ab, hw, kw, ti, ot, tx, ct, sh, tn, dm, mf, dv, fx, dq, nm, kf, ox, px, rx, an, ui, sy]<br>21. novel coronavirus 2019.mp. [mp=ab, hw, kw, ti, ot, tx, ct, sh, tn, dm, mf, dv, fx, dq, nm, kf, ox, px, rx, an, ui, sy]<br>22. Whuan coronavirus.mp. [mp=ab, hw, kw, ti, ot, tx, ct, sh, tn, dm, mf, dv, fx, dq, nm, kf, ox, px, rx, an, ui, sy]<br>23. Whuan pneumonia.mp. [mp=ab, hw, kw, ti, ot, tx, ct, sh, tn, dm, mf, dv, fx, dq, nm, kf, ox, px, rx, an, ui, sy]<br>24. Sars-CoV-2.mp. [mp=ab, hw, kw, ti, ot, tx, ct, sh, tn, dm, mf, dv, fx, dq, nm, kf, ox, px, rx, an, ui, sy]<br>25. Severe acute respiratory syndrome.mp. [mp=ab, hw, kw, ti, ot, tx, ct, sh, tn, dm, mf, dv, fx, dq, nm, kf, ox, px, rx, an, ui, sy]<br>26. 16 or 17 or 18 or 19 or 20 or 21 or 22 or 23 or 24 or 25<br>27. 15 and 26<br>28. limit 27 to yr="2019 -Current"	Conducted up to June 28th, 2021.

Table S2. List of studies excluded at full-text screening stage with brief reasons

Autor	Year	Journal paper?	Full text available?	1. Prospective cohort?	Reason 1	2. Population?	Reason 2	3. Outcome?	Reason 3	Comments
Abbas	2020			N	Cross-sectional/ Retrospective study					
Abdulaal	2020			N	Retrospective study					
Abohamr	2020			N	Descriptive case series					
Abolghasemi	2020	Letter				N	No inception cohort			
Abrahim	2020									
Abrishami	2020					N	No inception cohort			
Aguila-Gordo	2021			N	Retrospective study					
Ali	2020			N	Retrospective study					
Alisadehsani	2020					N	Included diagnosis for CT			
Allameh	2020			N	Retrospective study					
Allwood	2020	Protocol								
Al-Salameh	2020			N	Retrospective study					
Al-Salameh	2021			N	Retrospective study					
Alser	2021	Conference Abstract								
Al-Sulaiman	2021					N	No inception cohort			
Alvarez-Maldonado	2021					N	No inception cohort			
Andres-Esteban	2021					N	No inception cohort			
Apea	2021					N	Included people <18 y			
Arentz	2020	Letter								
Aries	2021		N							
Arnold	2020					N	No inception cohort			
Asch	2020					N	No inception cohort			
Auld	2020			N	Retrospective study					
Aw	2020					N	Included diagnosis for CT			
Aydin	2020					N	Included diagnosis for CT			

**Bahier**	2020					N	Included no hospitalized patients			
**Baiardo Redaelli**	2021					N	No inception cohort			
**Baktash**	2020					N	Included diagnosis for x-ray			
**Baqi**	2021			N	Retrospective study					
**Baumer**	2020					N	Inluded suspected or confirmed COVID-19			
**Bayrak**	2021					N	No inception cohort			
**Beccheti**	2020					N	No inception cohort			
**Bels**	2020					N	Included diagnosis for CT			
**Berenguer**	2021			N	Retrospective study					
**Beunier**	2020					N	Included diagnosis for CT and clinical symptoms			
**Bezerra**	2021			N	Retrospective study					
**Bezzio**	2020					N	Included diagnosis for CT and clinical symptoms			
**Bloom**	2020					N	No inception cohort			
**Booth**	2020						Included clinical and radiological diagnosis			
**Bousquet**	2020					N	Included radiological diagnosis			
**Bradley**	2020					N	No inception cohort			
**Brar**	2020			N	Retrospective study					
**Brenner**	2020					N	Included people <18 y			
**Brieghel**	2021			N	Retrospective study					
**Budhi**	2020	Conference Abstract								
**Burrel**	2020					N	Included people <18 y			
**Cagnazzo**	2020					N	No inception cohort			
**Calik Basaran**	2021					N	Included non confirmed COVID-19			
**Calvo-Fernandez**	2020					N	No inception cohort			
**Cariou**	2020					N	Included clinical/radiological diagnosis			

**Carter**	2020					N	Included clinical/radiological diagnosis			
**Chaibi**	2020			N	Retrospective study					
**Chen**	2020			N	Retrospective study					
**Chew**	2021			N	Retrospective study					
**Ciceri**	2020					N	Included radiological diagnosis			
**Coca**	2020					N	No inception cohort			
**Conway**	2020					N	No inception cohort			
**Covino**	2021					N	No inception cohort			
**Crespo**	2020					N	Included no hospitalized people			
**Daifi**	2020			N	Retrospective study					
**Dang**	2020	Conference Abstract								
**De Roubin**	2021			N	Retrospective study					
**De Souza**	2021					N	Included people <18 y			
**de-Azambuja**	2020					N	Included diagnosis for CT and clinical symptoms			
**Dobre**	2021					N	No inception cohort			
**Doyen**	2020					N	No inception cohort			
**Drake**	2020			N	Retrospective study					
**Du**	2020					N	Included clinical diagnosis			
**Dushimova**	2020	Conference Abstract								
**Elhadi**	2021					N	No inception cohort			
**Elkrief**	2020					N	No inception cohort			
**El-Solh**	2021			N	Retrospective study					
**Fadulelmola**	2020			N	Retrospective study					
**Farre**	2020					N	No inception cohort			
**Favalli**	2020					N	No inception cohort			
**Fenioux**	2021					N	No inception cohort			
**Fernandez-Capitan**	2020					N	No inception cohort			

**Fernandez-Capitan**	2021					N	No inception cohort			
**Ferrando-Vivas**	2021					N	No inception cohort			
**Figueroa-Pena**	2020					N	No inception cohort			
**Flaatten**	2021					N	No inception cohort			
**Fominskiy**	2021					N	Included diagnosis for CT and clinical symptoms			
**Forest**	2020			N	Retrospective study					
**Frager**	2020					N	No inception cohort			
**Fried**	2020					N	No inception cohort			
**Fried**	2021					N	No inception cohort			
**Frontera**	2020					N	No inception cohort			
**Fuentes Antras**	2020					N	No inception cohort			
**Gadotti**	2020					N	Included inception > 10 days of onset symptoms			
**Garcia-Vidal**	2021					N	No inception cohort			
**Gavrilina**	2020					N	No inception cohort			
**Georges**	2020			N	Clinical trial					
**Geretti**	2020					N	No inception cohort			
**Gessler**	2021					N	No inception cohort			
**Ghio**	2020			N	Retrospective study					
**Giacomelli**	2021									Duplicate data
**Gil-Rodrigo**	2020					N	Included clinical diagnosis			
**Glasbey**	2020					N	No inception cohort			
**Glenthoj**	2020					N	Included no hospitalized people			
**Haase**	2020			N	Retrospective study					
**Haberman**	2020					N	Included non confirmed COVID-19			
**Hagg**	2020					N	Included non confirmed COVID-19			
**Hayek**	2020					N	No inception cohort			
**Heberto**	2020					N	No inception cohort			
**Hernandez-Rubio**	2020					N	No inception cohort			
**Hewitt**	2020					N	Included clinical diagnosis			
**Hicini**	2020					N	Included assymptomatics people			

**Hirsch**	2020			N	Retrospective study					
**Hittersdof**	2020			N	Retrospective study					
**Hollein**	2020	Letter								
**Iftimie**	2020					N	No inception cohort			
**Inciardi**	2020					N	No inception cohort			
**Jaspard**	2021					N	Included people <18 y			
**Karbalai**	2020			N	Retrospective study					
**Kasivisvanathan**	2020					N	Patients no hospitalized by COVID-19			
**Kates**	2020					N	No inception cohort			
**Ken-Dror**	2020					N	No inception cohort			
**Khalil**	2020					N	No inception cohort			
**Khalili**	2020					N	No inception cohort			
**Khan**	2020			N	Retrospective study					
**Khan**	2020					N	No inception cohort			
**Khan**	2020					N	No inception cohort			
**Kirenga**	2020					N	Included people <18 y			
**Kjeldsen**	2021					N	No inception cohort			
**Knopp**	2020					N	No inception cohort			
**Knox**	2021					N	No inception cohort			
**Kocay**	2020					N	No inception cohort			
**Kocayigit**	2021					N	No inception cohort			
**Kottlors**	2020			N	Retrospective study					
**Kragholm**	2020					N	No hospitalized people			
**Kristinsson**	2020					N	No inception cohort			
**Kundi**	2020					N	No inception cohort			
**Kurtz**	2021					N	No inception cohort			
**Laake**	2021					N	No inception cohort			
**Lambermont**	2021					N	No inception cohort			
**Laosa**	2020					N	No inception cohort			
**Lassen**	2020					N	No inception cohort			
**Lee**	2020			N	Retrospective study					
**Li**	2020			N	Retrospective study					
**Li**	2020			N	Retrospective study					
**Li**	2021					N	No inception cohort			

**Liu**	2020					N	No hospitalized people			
**Liu**	2020					N	No inception cohort			
**Long**	2020					N	No inception cohort			
**Lopez-Otero**	2020					N	No inception cohort			
**Lowe**	2021			N	Retrospective study					
**Ma**	2020					N	No inception cohort			
**Mandel**	2020					N	No inception cohort			
**Manocha**	2020			N	Retrospective study					
**Marcolino**	2021			N	Retrospective study					
**Marengoni**	2020			N	Retrospective study					
**Martin-Sanchez**	2020			N	Retrospective study					
**Mato**	2020					N	No inception cohort			
**Mattar**	2020					N	Patients no hospitalized by COVID-19			
**Mehta**	2020					N	Included assymptomatics people			
**Mendizabal**	2020					N	Included assymptomatics people			
**Mendizabal**	2020	Conference Abstract								
**Messika**	2020			N	Retrospective study					
**Miles**	2020					N	Included non confirmed COVID-19			
**Mithal**	2020					N	No inception cohort			
**Mody**	2021					N	No inception cohort			
**Munoz**	2020			N	Retrospective study					
**Nabors**	2021		N							
**Nadeen**	2020	Letter								
**Nemer**	2020					N	No inception cohort			
**Nemer**	2021			N	Retrospective study	N	No inception cohort			
**Nunez-Gil**	2020					N	No inception cohort			
**Ozer**	2021			N	Retrospective study					
**Patel**	2021					N	No inception cohort			
**Peng**	2021			N	Retrospective study					
**Perez-Segura**	2021			N	Retrospective study					

**Peterlana**	2020	Conference abstract								
**Petrilli**	2020					N	No inception cohort			
**Petrulewicz**	2020					N	No inception cohort			
**Pinto**	2020					N	No inception cohort			
**Plotnikow**	2020					N	No inception cohort			
**Potalivo**	2020					N	No inception cohort			
**Price**	2020	Protocol								
**Raad**	2020	Conference abstract								
**Rahim**	2020			N	Cross-sectional study					
**Richards-Belle**	2020					N	No inception cohort			
**Robinson**	2020					N	No inception cohort			
**Rodriguez-Lago**	2020					N	No inception cohort			
**Rubio-Rivas**	2020			N	Retrospective study					
**Sachdeva**	2020					N	No inception cohort			
**Salvador**	2021					N	No inception cohort			
**Sami**	2020					N	No inception cohort			
**Santus**	2020					N	No inception cohort			
**Sarfaraz**	2021					N	Included assymptomatic people			
**Savasi**	2020					N	Included assymptomatics people			
**Seiglie**	2020					N	No inception cohort			
**Serling-Boyd**	2020					N	Included no hospitalized people			
**Sharma**	2021					N	Included people <18 y			
**Shwerzmann**	2021					N	Included diagnosis for CT and clinical symptoms			
**Skorin**	2020					N	No inception cohort			
**Smati**	2020			N	Retrospective study					
**Snipelilisk**	2020		N							
**Stockenhuber**	2020					N	No inception cohort			
**Sy**	2020					N	Included no hospitalized people			
**Tadolini**	2020					N	No inception cohort			
**Taramasso**	2020			N	Retrospective study					
**Tatum**	2020					N	No inception cohort			
**Tehrani**	2020			N	Retrospective study					

**Tomasoni**	2020			N	Retrospective study					
**Trabulus**	2020					N	No inception cohort			
**Vaid**	2020			N	Retrospective study					
**Vizcarra**	2020					N	Included suspected cases of COVID-19			
**Vlachos**	2021					N	Data on symptom onset were missing			
**Vrsalovic**	2020	Letter								
**Vuagnat**	2020					N	Included non hospitalized people			
**Wang**	2020					N	No inception			
**Wang**	2020			N	Retrospective study					
**Wang**	2021					N	Included people <18 y			
**Webb**	2020					N	Included non hospitalized people			
**Wendel**	2020					N	Included people <18 y			
**Yang**	2020					N	No inception cohort			
**Yehia**	2020			N	Retrospective study					
**Zeng**	2020			N	Retrospective study					
**Zhang**	2020					N	Diagnosis not described			
**Zhang**	2020			N	Retrospective study					
**Zhang**	2021			N	Retrospective study					
**Zhao**	2020			N	Retrospective study					
**Zinellu**	2020			N	Retrospective study					
**Zorzi**	2020			N	Retrospective study					
**Zuccon**	2020			N	Retrospective study					

Abbreviations: N=Not; CT=computed tomography.

Table S3. Table of characterization

Study	Country	Sample size	Age Mean (SD) or Median (IQR)	Sex No (%)	Hospital setting	Comorbidities No (%)	Onset of symptoms Days median (IQR)	Outcomes No (%) Days median (IQR)
Allenbach et al., (2020)[20]	France	152	77 (60–83)	Male 91 (59.9)<br>Female 61 (40,13)	Hospital<br>(17 to ICU)	Smoker 10 (6.6), HBP 82 (53.9), DM 37 (24.3), DLP 50 (32.9), IHD 35 (23), Cancer 30 (19.7), COPD 12/151 (7.9)	5 (2-8)	**Mortality:** 32 (21.9), 14 days<br>**Recovery:** 84 (57.5)
Barrasa et al. (2020)[34]	Spain	48	63 (51–75)	Male 27 (56)<br>Female 21 (44)	ICU	Obesity 23 (48), HBP 21 (44), DM 9 (19), Smoker 9 (19), HT 9 (19), HD 5 (10), Immunosuppression 3 (6), COPD 18 (38), solid organ transplant 1 (2)	7 (5–12)	**Mortality:** 6 (13) 7 days, 14 (31), 15 days
Brandão et al. (2021)[14]	Brazil	506	59.2 (±16.8)	Male 290 (57.3)<br>Female 216 (42.7)	Hospital<br>(300 to ICU)	HBP 280 (55.3%); diabetes 181 (35.8); smoking history 144 (39.2%); renal replacement therapy 15 (3.0%); Congestive heart failure 39 (7.7%); COPD 15 (3.0%); asthma 22 (4.4%); Other lung diseases 12 (2.4%); cancer 39 (7.7%); solid organ transplant 16 (3.2%); systemic lupus erythematosus 5 (1.0%); HIV 7 (1.4%).	<10	**Mortality**: 153 (30.2), 21 days<br>**Recovery:** 333 (65.9)
Cao et al. (2020)[15]	China	102	54 (37–67)	Male 53 (52)<br>Female 49 (48)	Hospital<br>(18 to ICU)	DM 11 (10.8), HBP 28 (27.5), CVD 5 (4.9) CRD 10 (9.8), cerebrovascular disease 6 (5.9), malignancy 4 (3.9), CKD 4 (3.9), CLD 2 (2.0)	6 (3–7)	**Mortality:** 17 (16.6), 15 (9–21) days<br>**Morbidity:** Shock 10 (9.8), ARDS 20 (19.6)<br>**Recovery:** 85 (83.3)
Cortes-Telles et al. (2020)[22]	México	200	55 (41–65)	Male 138 (69)<br>Female 62 (31)	Hospital<br>(44 to ICU)	Obesity (52), HBP (30), DM (28)	8 (6–9)	**Mortality:** 77 (38), 8 (4–16)
Cummings et al. (2020)[43]	United States	257	62 (51–72)	Male 171 (67)<br>Female 86 (33)	ICU	DM 92 (36) HBP 162 (63), CHD 49 (19), COPD or ILD 24 (9), asthma 21 (8), smoking history 33 (13), chronic neurological disease or dementia 24 (9), cancer (7), CKD 37 (14), LC 5 (2), solid organ transplant recipient 10 (4), HIV 8 (3)	5 (2-7)	**Mortality:** 101 (39), 9 (5–15) days<br>**Recovery:** 58 (23)
Dzupová et al. (2021)[28]	Zech Republic	74	67.5 (56-75)	Male 46 (62.2)<br>Female 28 (37,8)	ICU	CRD 17 (23.0); COPD only 7 (9.5); immunodeficiency 6 (8.1); diabetes 30 (40.5); HBP 48 (64.9); CAD 16 (21.6); CKD 10 (13.5); CLD 5 (6.8); Cancer 7 (9.5); Obesity 61 (82,5)	6 (4–8)	**Mortality:** 30 (40.5)<br>**Morbidity:** ARDS 57 (77.0)<br>**Recovery:** 31 (41.9)
Estella et al. (2021)[33]	Spain	422	63 (54-71)	Male 305 (72.3)<br>Female 117 (27.7)	ICU	COPD 25 (5.9), CKD 44 (10.4), cirrhosis 11 (2.6), cancer 30 (7.1)	7 (4-9)	**Mortality**: 152 (36.5), 42 days
Ferrando et al. (2020)[38]	Spain and Andorra	663	64 (56-72)<br>No=661	Male 441 (66.7)<br>Female 219 (33.18)<br>No=660	ICU	HBP 329 (49.6), DM 151 (22.7), CHD 9 (1.3), CKD 38 (5.7), asthma 17 (2.5), COPD 28 (4.2), DLP 90 (13.5), cancer 23 (3.4)	7 (5-9)	**Mortality** 203 (30.6), 80 days<br>**Morbidity:** SDRA 601 (90.6), Shock 150 (22.6)

Giacomelli et al. (2020)[21]	Italy	233	61 (50–72)	Male 161 (69.1)<br>Female 72 (30.9)	Hospital and ICU (145 – mild and moderate, 88 – severe and critical)	Smoking history 70 (30), obesity 38 (16.3)	7 (4−9)	**Mortality:** 48 (20,6), 11 (6–18) days<br>**Recovery:** 162 (69.5)
Gupta et al. (2020)[41]	United States	2215	60.5 (14.5)	Male 1436 (64.8)<br>Female 779 (35.2)	ICU	DM 861 (38.9), HBP 1322 (59.7), CAD 288 (13.0), CHF 196 (8.8), CLD 531 (24.0), COPD 173 (7.8), asthma 258 (11.6), smoking history 656 (29.6), CKD 280 (12.6), end-stage KD 64 (2.9), malignancy 112 (5.1), immunodeficiency 65 (2.9)	7 (4-10)	**Mortality:** 875 (39.5); 16 (8-28)<br>**Morbidity:** Respiratory failure 727 (92.7), Septic shock 311 (39.7)<br>**Recovery:** 1.203 (54.3)
Jourdes et al. (2020)[18]	France	263	65 (54–76)	Male 155 (58.9)<br>Female 108 (41,06)	Hospital (111 to ICU)	Overweight 88/244 (36.1), obesity 69/244 (28.3) HBP 104 (39.5), DM 52 (19.8), HD 35 (13.3) cerebrovascular disease 17 (6.5), CRD 57 (21.6), CKD 24 (9.1), CLD 2 (0.8), malignancy 27 (10.3), immunosuppression 24 (9.1) smokers 11/115 (9.3)	7 (4 –10)	**Mortality:** 19, 14 days<br>**Recovery:** 173, 14 days
Kaeuffer et al. (2020)[19]	France	1045	66 (20–100)	Male 612 (59)<br>Female 433 (41,4)	Hospital (335 to ICU)	HBP 548 (52.4), DM 264 (25.3), smoker 36 (3.4), CHF 121 (11.6), CRD 172 (16.5), CKD 117 (11.2), CLD 11 (1.1), immunosuppression 48 4.6, cancer 109 (10.4), pregnancy 15 (1.4)	7.2 (5.3)	**Mortality:** 115 (11), 7 days, 195 (18.7), 30 days<br>**Morbidity:** 335 (32.1)<br>**Recovery:** 155 (14.8)
Kayina et al. (2020)[30]	India	235	50.7±15.1	Male 160 (68.1)<br>Female 75 (31,91)	ICU	HBP 65, DM 54, CAD 13, CKD 22, CLD 10, COPD 5, cancer 26	4 (2-5)	**Mortality:** 20, 1 day
Larsson et al. (2020)[39]	Sweden	260	59 (51-65)	Male 208 (80.0)<br>Female 52 (20)	ICU	HBP 103 (39.6), CHD 17 (6.5), COPD/asthma 33 (12.7), immunodeficiency 15 (5.8), CLD 1 (0.4), CKD 4 (1.5), DM 68 (26.2), neuromuscular disease 3 (1.2)	11 (8-14)	**Mortality:** 60, 12 days (6-18)<br>**Recovery:** 62 (23.9)
Madsen et al. (2021)[17]	Denmark	83	62 (54-74)	Male 52 (62.7)<br>Female 31 (37.3)	Hospital (13 transfer for ICU)	CVD 46 (55.4), HBP 35 (42.2), stroke 4 (4.8), CRD 14 (16.9), malignancy 14 (16.9), DM 13 (15.7), inflammatory bowel disease 3 (3.6), rheumatoid arthritis 3 (3.6), CKD 2 (2.4)	9 (5.5-11)	**Mortality:** 4, 10 days (6.5-13)<br>**Morbidity:** ARDS, 28 (33.7)<br>**Recovery:** 78 (94
Molnar et al. (2020)[42]	United States	386	60 (51-70)	Male 277 (72)<br>Female 109 (28)	ICU	DM 253 (66), HBP 317 (82), CAD 104 (27), CHF 71 (18), atrial fibrilllation or atrial flutter 48 (12), COPD 21 (5), asthma 23 (6), smoking history 117, CKD 198 (51), CLD 35 (9), HIV 10 (3), cancer 25 (6)	5 (3-8)	**Mortality:** 163, 28 days<br>**Morbidity:** ARDS, 52, Septic shock, 26
Myrstad et al. (2020)[24]	Norway	66	71.5 (30–95)	Male 38 (58)<br>Female 28 (42)	Hospital (7 transfer to ICU)	HBP 4 (27), DM 3 (20), CKD 2 (13), Malignancy 3 (20), COPD 4 (27)	6.9 (3.8)<br>8.7 (5.0)	**Mortality:** 13 (20), 5 days<br>**Recovery:** 53 (80)
Namendys-Silva et al. (2021)[32]	Mexico	164	57.3 (SD 13.7)	Male 114 (69.5)<br>Female 50 (30,48)	ICU	HBP 63 (38.4), DM 53 (32.3), smoker 24 (14.3), solid tumors 10 (6.0), COPD 4 (2.4), CKD 6 (3.6)	7 (4.5-9)	**Mortality:** 85 (51.8), 30 days

Olivas-Martínez et al. (2021)[23]	Mexico	800	51.9 (13.9)	Male 488 (61)<br>Female 312 (39)	Hospital<br>(199 transfer to ICU)	Diabetes 209 (26), obesity 357/797 (44.8), HBP 240 (30), CRD 7 (0.9), asthma 11 (1.4), immunosupression 48 (6), HIV 10 (1.2), CVD 37 (4.6), CKD 24 (3), CLD 6 (0.8), Smoking 112 (14)	8 (6-10)	**Mortality:** 241<br><br>**Morbidity:** ARDS 159 (67), MOFS 67
Rodríguez et al. (2020)[35]	Spain	43	65.5 (52-72)	Male 27 (62.8)<br>Female 16 (37.2)	ICU	DM 18 (18.6), HBP 13 (30.2), CHD 2 (4.7) IHD 4 (9.3), COPD 4 (9.3), CKD 2 (4.7), obesity 11 (25.6), autoimmune disease 3 (7)	8 (6-10)	**Mortality:** 10 (23.5), 28 days<br>**Morbidity:** ARDS 40 (93)<br>**Recovery:** 28 (60.5)
Pineiro et al. (2021)[36]	Spain	52	71.5 (61–74.7)	Male 40 (77)<br>Female 12 (23)	ICU	None 14 (26.9), HBP 31 (59.6), DM 11 (21.2), CHF 2 (3.8), IHD 3 (5.8), obesity 9 (17.3), COPD 11 (21.2), smoker 3 (5.8), cancer 3 (5.8), HIV 1 (1.9), CKD 7 (13.5)	10 (4)	**Mortality:** 27 (51.92), 35.5 (IQR 36.75)<br>**Recovery:** 25 (48)
Piwowarczyk et al. (2020)[25]	Poland	60	ARF group 59 (50–67), Non-ARF group 39 (25–51)	Male 31(51,66)<br>Female 29 (48,33)	Hospital<br>(10 in MV)	HBP 20, COPD 1, diabetes 4, CVD 8, LD 2, CKD 0	7 (4–8)	**Mortality:** 8<br>**Morbidity:** ARDS 22 (36.6)
Radovanovic et al. (2021)[31]	Italy	2nd p= 76	2nd p= 68 (55–77)	2nd p= Male 56 (74)<br>Female 20 (26)	ICU	2nd p= HBP 33 (43.4), diabetes 23 (30.3), IHD 17 (22.4), CHF 5 (6.6), arrythmia 7 (9.2), COPD 8 (10.5), asthma 8 (10.5), obesity 9 (11.8), psychiatric disorders 4 (5.3), cancer 7 (9.2), CKD 6 (7.9)	2nd p= 5 (6–13)	**Mortality:** 2nd p= 17 (22.4)<br>**Recovery:** 42 (55.4)
Roberts et al. (2020)[27]	United States	40	61 (52-66)	Male 28 (70)<br>Female 12 (30)	Hospital<br>(11 ICU)	HBP 33 (83), DM 15 (38), CKD 30 (75), CRD 8 (20), asthma 3 (8), HIV 1 (3), HF 9 (23), HLP 14 (35), cancer 1 (3), smoking history 19 (48)	7 (10)	**Mortality:** 5 (16), 10-28 days<br>**Recovery:** 23 (72)
Rodriguez-Molinero et al. (2020)[26]	Spain	418	65.4 (SD 16.6)	Male 238 (56,9)<br>Female 180 (43,1)	Hospital<br>(254 – severe disease)	CKD 61, HBP 217, DM 99, DLP 145, obesity 74, smoking 36, alcoholism 11, HF 26, IHD 37, atrial fibrillation 45, stroke 2, asthma 23, COPD 41, dementia 43, cancer 48, HIV 3, organ transplant 1	8.0 (SD 4.5)	**Mortality:** 79, 30 days
Schmidth et al. (2020)[29]	France, Switzerland, and Belgium	4244	63 (54–71)	Male 3159 (74.4)<br>Female 1085 (26)	ICU	HBP 2018 (48), DM 1167 (28), immunodeficiency 314 (7)	9 (6–12)	**Mortality:** 1.298, 90 days<br>**Morbidity:** ARDS 2233
Taboada et al. (2020)[37]	Spain	97	69 (61-73)	Male 62 (63.9)<br>Female 35 (36.08)	ICU	HBP 55 (56.7), DLP 42 (43.3), DM 22 (22.7), asthma 7 (7.2), COPD 11 (11.3), HD 28 (28.9), cancer 7 (7.2), obesity 38 (39.2)	10 (7-12)	**Mortality:** 15, 42 (34-45) days
Thomson et al. (2020)[40]	England	156	62 (54-70)	Male 112 (72)<br>Female 44 (28)	ICU	HBP 81, DLP 56, DM 52, IHD 26, CKD 23, CRD 19, end-stage renal failure on dialysis 13	7 (5-10)	**Mortality:** 38, 30 days<br>**Recovery:** 82
Yang et al. (2021)[16]	China	357	56.0 (43.0, 68.0)	Male 185 (51.8)<br>Female 172 (48,1)	Hospital	Coexisting disorders 154 (43.1), (including HBP, DM, CRD, CVD or cerebrovascular disease, CKD, HIV metastatic malignancy, CLD)	9.0 (5.0, 15.0)	**Mortality:** 25, 28 days

Abbreviations: ICU=intensive care unit; ARF=acute respiratory failure; ARDS=acute respiratory distress syndrome; 2nd p= second period; HBP=high blood pressure; DM=diabetes mellitus; DLP=dyslipidemia; IHD=ischemic heart disease; COPD=chronic obstructive pulmonary disease; HT=hypothyroidism; HD=heart disease; CVD=cardiovascular disease; CHF=chronic heart failure; CRD=chronic respiratory diseases; CKD=chronic kidney disease; CLD=chronic liver disease; ILD=interstitial lung disease; LC=liver cirrhosis; HIV=human immunodeficiency virus; CAD=coronary artery disease; KD=kidney disease; HLP=hyperlipidemia; SD=standard deviation; IQR=interquartile range.

Table S4. Distribution of patients for setting and outcomes prevalence.

Setting	No	Transfer to ICU/ MV	Mortality No	Morbidity MOFS No	ARDS No	Recovery No
Hospital ward	4325	1407	916	412/2,869	229/ 1,045	991/ 1,445
ICU	9392	-	3,457	487/3,264	3,710/ 7,625	1,531/3,133
Total	13,717	1407	4,373	899/6,133	3,939/ 8,670	2,522/ 4,578

Abbreviations: ICU=intensive care unit; MV=mechanical ventilation; MOFS=multiorgan failure syndrome; ARDS=acute respiratory distress syndrome.

Figure S1. Prevalence of hypertension in the sample.

Country	Hospitalized / Hospital ward	Sample size	Events	Proportion (95% CI)
Brazil	Brandão et al.[14]	506	280	0·55 (0·51-0·59)
China	Cao et al.[15]	102	28	0·27 (0·19-0·36)
Denmark	Madsen et al.[17]	83	35	0·42 (0·32-0·53)
France	Allenbach et al.[20]	152	82	0·53 (0·46-0·61)
France	Jourdes et al.[18]	263	104	0·39 (0·33-0·45)
France	Kaeuffer et al.[19]	1045	548	0·52 (0·49-0·55)
Mexico	Cortes-Telles et al.[22]	200	30	0·15(0·10-0·20)
Mexico	Olivas-Martínez et al.[23]	800	240	0·30 (0·26-0·33)
Norway.	Myrstad et al.[24]	66	04	0·06 (0·02-0·15)
Poland .	Piwowarczyk et al.[25]	60	20	0·33 (0·22-0·46)
Spain	Rodriguez-Molinero et al.[26]	418	217	0·51 (0·47-0·56)
USA	Roberts et al.[27]	40	33	0·82 (0·67-0·91)
	**ICU**			
Czech Republic	Dzupová et al.[28]	74	48	0·64 (0·53-0·74)
France, Switzerland, Belgium	Schmidth et al.[29]	4244	2018	0·47 (0·46-0·49)
India	Kayina et al.[30]	235	65	0·27 (0·22-0·33)
Italy	Radovanovic et al.[31]	76	33	0·43 (0·32-0·54)
Mexico	Namendys-Silva et al.[32]	164	63	0·38 (0·31-0·46)
Spain	Barrasa et al.[34]	48	21	0·43 (0·30-0·57)
Spain	Rodríguez et al.[35]	43	13	0·30 (0·18-0·45)
Spain	Pineiro et al.[36]	52	31	0·59 (0·45-0·72)
Spain	Taboada et al.[37]	97	55	0·56 (0·46-0·66)
Spain and Andorra	Ferrando et al.[38]	663	329	0·49 (0·45-0·53)
Sweden	Larsson et al.[39]	260	103	0·39 (0·33-0·45)
UK	Thomson et al.[40]	156	81	0·51 (0·44-0·59)
USA	Gupta et al.[41]	2215	1322	0·59 (0·57-0·61)
USA	Molnar et al.[42]	386	317	0·82 (0·78-0·85)
USA	Cummings et al.[43]	257	162	0·63 (0·57-0·68)

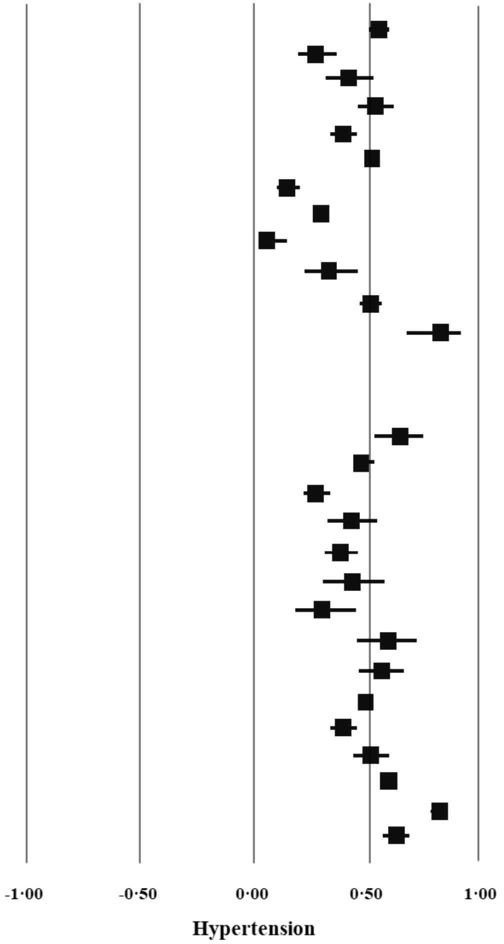

For analysis, the studies were grouped by country and by hospital setting (hospital ward or ICU). Estimates of proportions for dichotomous data, considering number of events and sample size, were reported. Abbreviations: ICU=intensive care unit; CI=confidence interval; USA=United States of America; UK=United Kingdom.

Figure S2. Prevalence of diabetes in the sample.

Country	Hospitalized	Sample size	Events	Proportion (95% CI)
	**Hospital ward**			
Brazil	Brandão et al.[14]	506	181	0·35 (0·31-0·40)
China	Cao et al.[15]	102	11	0·10 (0·06-0·18)
Denmark	Madsen et al.[17]	83	13	0·15 (0·09-0·25)
France	Allenbach et al.[20]	152	37	0·24 (0·18-0·31)
France	Jourdes et al.[18]	263	52	0·19 (0·15-0·25)
France	Kaeuffer et al.[19]	1045	264	0·25 (0·22-0·28)
Mexico	Cortes-Telles et al.[22]	200	28	0·14 (0·09-0·19)
Mexico	Olivas-Martínez et al.[23]	800	209	0·26 (0·23-0·29)
Norway	Myrstad et al.[24]	66	03	0·04 (0·01-0·13)
Poland	Piwowarczyk et al.[25]	60	04	0·06 (0·02-0·16)
Spain	Rodriguez-Molinero et al.[26]	418	99	0·23 (0·19-0·28)
USA	Roberts et al.[27]	40	15	0·37 (0·24-0·53)
	**ICU**			
Czech Republic	Dzupová et al.[28]	74	30	0·40 (0·30-0·52)
France, Switzerland, Belgium	Schmidth et al.[29]	4244	1167	0·27 (0·26-0·28)
India	Kayina et al.[30]	235	54	0·23 (0·18-0·28)
Italy	Radovanovic et al.[31]	76	23	0·30 (0·21-0·41)
Mexico	Namendys-Silva et al.[32]	164	53	0·32 (0·25-0·39)
Spain	Barrasa et al.[34]	48	09	0·18 (0·10-0·32)
Spain	Rodríguez et al.[35]	43	18	0·41 (0·28-0·56)
Spain	Pineiro et al.[36]	52	11	0·21 (0·12-0·34)
Spain	Taboada et al.[37]	97	22	0·22 (0·15-0·32)
Spain and Andorra	Ferrando et al.[38]	663	151	0·22 (0·19-0·26)
Sweden	Larsson et al.[39]	260	68	0·26 (0·21-0·31)
UK	Thomson et al.[40]	156	52	0·33 (0·26-0·41)
USA	Gupta et al.[41]	2215	861	0·38 (0·36-0·40)
USA	Molnar et al.[42]	386	253	0·65 (0·60-0·70)
USA	Cummings et al.[43]	257	92	0·35 (0·30-0·41)

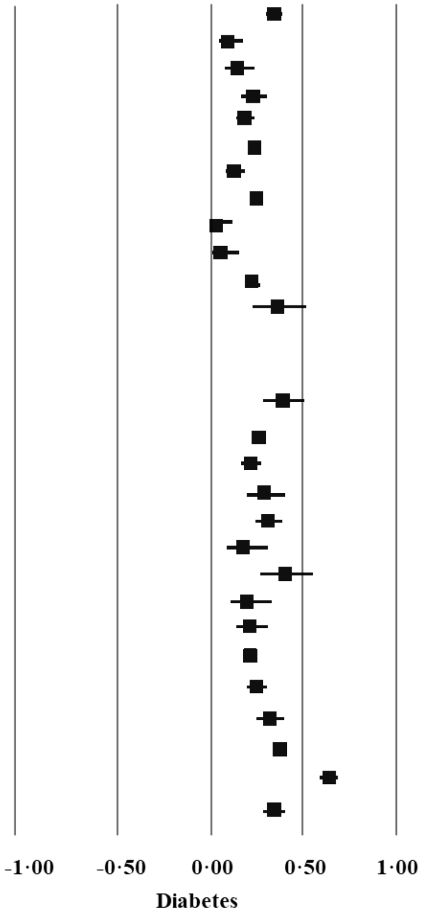

For analysis, the studies were grouped by country and by hospital setting (hospital ward or ICU). Estimates of proportions for dichotomous data, considering number of events and sample size, were reported. Abbreviations: ICU=intensive care unit; CI=confidence interval; USA=United States of America; UK=United Kingdom.

Figure S3. **Prevalence of obesity in the sample.**

Country	Hospitalized	Sample size	Events	Proportion (95% CI)
	**Hospital ward**			
France	Jourdes et al.[18]	244	69	0·28 (0·23-0·34)
Italy	Giacomelli et al.[21]	233	38	0·16 (0·12-0·21)
Mexico	Cortes-Telles et al.[22]	200	52	0·26 (0·20-0·32)
Mexico	Olivas-Martínez et al.[23]	797	357	0·44 (0·41-0·48)
Spain	Rodriguez-Molinero et al.[26]	418	74	0·17 (0·14-0·21)
	**ICU**			
Czech Republic	Dzupová et al.[28]	74	61	0·82 (0·72-0·89)
France, Switzerland, Belgium	Schmidth et al.[29]	4244	38	0·00 (0·00-0·01)
Italy	Radovanovic et al.[31]	76	09	0·11 (0·06-0·21)
Spain	Barrasa et al.[34]	48	23	0·47 (0·34-0·61)
Spain	Rodríguez et al.[35]	43	11	0·25 (0·14-0·40)
Spain	Pineiro et al.[36]	52	09	0·17 (0·09-0·30)
Spain	Taboada et al.[37]	97	38	0·39 (0·30-0·49)

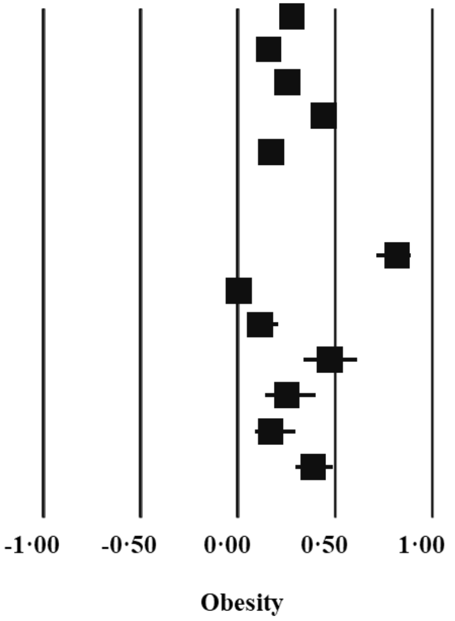

For analysis, the studies were grouped by country and by hospital setting (hospital ward or ICU). Estimates of proportions for dichotomous data, considering number of events and sample size, were reported. Abbreviations: ICU=intensive care unit; CI=confidence interval.

Figure S4. Prevalence of smoking history in the sample.

Country	Hospitalized<br>Hospital ward	Sample size	Events	Proportion (95% CI)
Brazil	Brandão et al.[14]	506	144	0·28 (0·24-0·32)
France	Jourdes et al.[18]	115	11	0·09 (0·05-0·16)
France	Kaeuffer et al.[19]	1045	36	0·03 (0·02-0·04)
Mexico	Olivas-Martínez et al.[23]	800	112	0·14 (0·11-0·16)
Italy	Giacomelli et al.[21]	233	70	0·30 (0·24-0·36)
Spain	Rodriguez-Molinero et al.[26]	418	36	0·08 (0·06-0·11)
USA	Roberts et al.[27]	40	19	0·47 (0·32-0·62)
	**ICU**			
Spain	Barrasa et al.[34]	48	09	0·18 (0·10-0·32)
Spain	Pineiro et al.[36]	52	03	0·05 (0·01-0·16)
USA	Gupta et al.[41]	2215	656	0·29 (0·27-0·31)
USA	Molnar et al.[42]	386	117	0·30 (0·25-0·35)
USA	Cummings et al.[43]	257	33	0·12 (0·09-0·17)

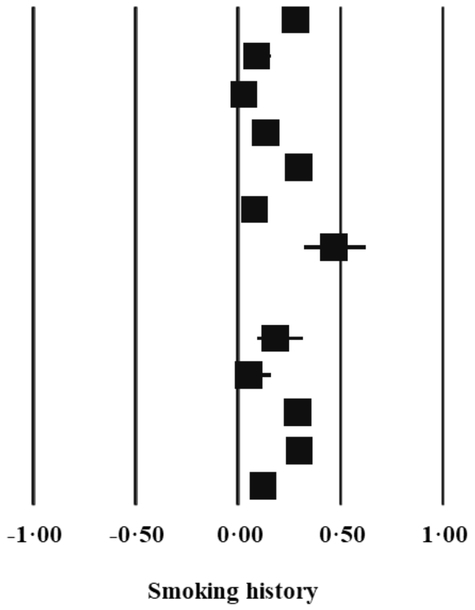

For analysis, the studies were grouped by country and by hospital setting (hospital ward or ICU). Estimates of proportions for dichotomous data, considering number of events and sample size, were reported. Abbreviations: ICU=intensive care unit; CI=confidence interval; USA=United States of America.

Table S5. Risk of bias assessment (QUIPS)

Study	Study Participation	Study Attrition	Prognostic Factor Measurement	Outcome Measurement	Study Confounding	Statistical Analysis and Reporting
Allenbach et al.[20]	Moderate	Low	N/A	Low	Moderate	Low
Barrasa et al.[34]	Moderate	Low	N/A	Low	High	Moderate
Brandão et al.[14]	Moderate	Low	N/A	Low	Moderate	Low
Cao et al.[15]	Moderate	Low	N/A	Moderate	High	Moderate
Cortes-Telles et al.[22]	Moderate	Low	N/A	Moderate	Moderate	Low
Cummings et al.[43]	Moderate	Moderate	N/A	Low	Moderate	Low
Dzupová et al.[28]	Low	Low	N/A	Moderate	High	Moderate
Estella et al.[33]	Low	Moderate	N/A	Low	Moderate	Low
Ferrando et al.[38]	Low	Moderate	N/A	Moderate	High	Low
Giacomelli et al.[21]	Moderate	Low	N/A	Low	Moderate	Low
Gupta et al.[41]	Moderate	Low	N/A	Low	Moderate	Low
Jourdes et al.[18]	Moderate	Low	N/A	Low	High	Moderate
Kaeuffer et al.[19]	Moderate	Low	N/A	Low	Moderate	Low
Kayina et al.[30]	Moderate	Moderate	N/A	Low	High	Moderate
Larsson et al.[39]	Moderate	Low	N/A	Low	Moderate	Low
Madsen et al.[17]	Low	Low	N/A	Low	High	Moderate
Molnar et al.[42]	Moderate	Low	N/A	Low	High	Moderate
Myrstad et al.[24]	Moderate	Low	N/A	Low	High	Low
Namendys-Silva et al.[32]	Moderate	Moderate	N/A	Low	Moderate	Low
Olivas-Martinez et al.[23]	Moderate	Moderate	N/A	Low	Moderate	Low
Rodríguez et al.[35]	Moderate	Low	N/A	Low	High	Moderate
Pineiro et al.[36]	Low	Low	N/A	Low	Moderate	Low
Piwowarczyk et al.[25]	Low	Low	N/A	Moderate	High	Low
Radovanovic et al.[31]	Moderate	Low	N/A	Moderate	High	Low
Roberts et al.[27]	Moderate	Low	N/A	Low	High	Moderate
Rodriguez-Molinero et al.[26]	Moderate	Low	N/A	Low	Moderate	Low
Schmidt et al.[29]	Moderate	High	N/A	Low	Moderate	Low
Taboada et al.[37]	Moderate	Low	N/A	Low	High	Moderate
Thomson et al.[40]	Moderate	Low	N/A	Low	Moderate	Low
Yang et al.[16]	Moderate	Low	N/A	Low	High	Low

Low: low risk of bias, Moderate: moderate risk of bias, High: high risk of bias. N/A: not applicable. The risk of bias was determined by judging the warming items for each domain.
